# Hydrophilic Dogwood Extracts as Materials for Reducing the Skin Irritation Potential of Body Wash Cosmetics

**DOI:** 10.3390/molecules22020320

**Published:** 2017-02-19

**Authors:** Zofia Nizioł-Łukaszewska, Paweł Osika, Tomasz Wasilewski, Tomasz Bujak

**Affiliations:** 1 Department of Cosmetology, The University of Information Technology and Management in Rzeszow, Kielnarowa 386a, Tyczyn 36-020, Poland; zniziol@wsiz.rzeszow.pl (Z.N.-Ł.); posika@wsiz.rzeszow.pl (P.O.); 2 Department of Chemistry, University of Technology and Humanities in Radom, Chrobrego 27, Radom 26-600, Poland; tomasz.wasilewski@uthrad.pl

**Keywords:** *Cornus mas*, extract, antioxidant activity, irritation potential, cosmetic safety

## Abstract

A significant problem related to the use of surfactants in body wash cosmetics is their propensity to trigger skin irritations. Only scarce literature exists on the effect of plant extracts on the skin irritation potential. The present study is an attempt to determine the effect of hydrophilic dogwood extracts on the irritant potential of body wash gels. Extractants used in the study were water and mixtures of water with glycerine, water with trimethylglycine (betaine), and water with plant-derived glycol (propanediol). The basic biochemical properties, i.e., the ability to neutralize free radicals, and the content of polyphenols, anthocyanins and flavonoids, were determined. An attempt was undertaken to analyze the impact of the extract added to natural body wash gel formulations on product properties. The skin irritation potential was assessed by determining the zein number and the increase in the pH level of the bovine serum albumin (BSA) solution. The viscosity and foaming ability of the resulting products were evaluated. The studies revealed that an addition of dogwood extract contributes to an improvement in the properties of body wash gels and significantly increases the safety of product use through reducing the skin irritation effect.

## 1. Introduction

The potential to trigger skin irritations is one of the greatest disadvantages of using body wash cosmetics. Substances responsible for the detergent activity constitute the basic ingredient in this group of cosmetic products. Their content in body wash formulations varies in the range of 10%–20% [1,2,3,4].

Washing agents are classified as belonging to the group of surfactants (surface-active agents), i.e., substances, the molecules of which contain two parts differing in polarity: hydrophilic (having an affinity to water) and hydrophobic (having an affinity to non-polar substances). The bipolar structure of molecules determines a range of surfactant properties. From the viewpoint of skin washing and skin irritation effects, the key property of surfactants is their adsorption capacity at phase interfaces [2,3,4,5]. Skin irritation can arise from various types of surfactant interaction with the skin. Based on the literature data [1,2,6,7,8,9,10,11,12,13,14,15,16], the primary mechanism of triggering skin irritations is the interaction of surfactants with surface proteins in the stratum corneum. Binding to proteins can result in the degradation of their structure and denaturation. The literature reports [6,7,8,9,10,11,12,13,14,15,16] also show that the highest propensity to cause skin irritations via the mechanism of interaction with peptides is associated with anionic surfactants such as sodium lauryl sulfate [7,8,10,11,12,14]. The reason is that anionic surfactants bind to protein chains through relatively strong electrostatic interactions. Other groups of surfactants interact with proteins via weak hydrogen bonds, which is why their skin irritation potential is insignificant [1,2]. Another proposed mechanism for triggering skin irritations involves the ability of surface-active agents to solubilize lipid substances and remove them from the skin. The process can result in the disruption of the liquid crystal structure of the intercellular cement and, as a consequence, impair the function of the skin as a protective barrier against the penetration of physical and chemical agents, and disease-causing pathogens [1,6,7,8,9,10,11,12,13,14,15,16]. Solubilization and disruption of the liquid crystal structure furthermore leads to an increase in transepidermal water loss (TEWL) [1,2,3,16]. Damage to the epidermal barrier allows the penetration of surfactants into deeper skin layers and their interactions with living cells. The mechanism represents the final factor, as reported in the literature, potentially leading to surfactant-induced skin irritation and allergy [1,3].

The ability of surfactants to trigger skin irritations is mainly attributable to their molecular structure, the concentration at which they are used and the associated property of the form in which they occur in solutions [3,6,7,8,9,10,11,12,13,14,15,16]. The highest skin irritation potential is ascribed to surfactants occurring in the form of monomers, i.e., individual molecules [1,2,3,4,5,6,7,8,9,10,11,12,13,14,15,16]. Due to the small monomer size, surfactants are able to penetrate easily into the skin and interact with proteins and the intercellular cement. Micelles formed in surfactant solutions when the critical micelle concentration (CMC) is exceeded are considerably larger in size [1,2,3]. The irritant potential of surfactants in the form of micellar aggregates is substantially lower [3,6,7,8,9,10]. There are multiple methods of reducing the skin irritation potential of surface-active agents. In industrial practice, the most common method is to use mixtures of various surfactant groups in body wash cosmetic formulations. It follows from the literature data [1,3,4] that adding a nonionic, amphoteric or cationic surfactant to a system containing an anionic surfactant results in an increase in the size of the forming micelles. Large micelles are unable to penetrate deep into the skin as easily. Consequently, their potential to trigger skin irritations becomes significantly reduced. Moreover, research demonstrates that a decrease in the irritant potential can be achieved through the introduction into a surfactant solution of synthetic and natural polymers, hydrolyzed proteins, hydrophilic solvents and electrolytes. By reducing the CMC, these substances decrease the amount of monomers in the solution [1,2,3,7,8,16,17,18,19,20].

There is ongoing search for new methods of lowering the irritant potential of surfactants. Only scarce literature exists on the effect of plant extracts on the surfactant-induced skin irritation potential. Katsarou et al. [21] indicate that plant extracts are a valuable source of natural antioxidants which, by neutralizing free radicals, can delay the process of decomposition of unsaturated lipids contained in the intercellular cement and thus protect against the impairment of the epidermal barrier, ultimately reducing the likelihood of skin irritations. In addition, active ingredients contained in extracts are capable of protecting enzymes found in the skin against surfactant-induced denaturation [22,23,24,25]. As regards the effect of extracts on the irritant potential of washing agents, the literature reports show that hydrophobic plant extracts obtained under supercritical CO_2_ conditions contribute to a decrease in the skin irritation effect of hand dishwashing liquids [20].

The present study attempted to determine the effect of hydrophilic dogwood extracts on the irritant potential of body wash gels. Multiple studies have shown that extracts and plant substances derived from *Cornus mas* contain significant amount of phenolic compounds, vitamin C, iridoids, flavonoids and, in particular, anthocyanins [26,27,28,29,30,31,32,33,34,35,36,37,38,39] which are largely attributable to their health promoting properties. The bioactive compounds from the family of phenolic acids such as elagic acid, *p*-cumaroylhhexoside and chlorogenic acid and compunds from the family of flavonols—quercitin-3-*O*-glucuronide, kaempferol-3-*O*-galactoside, identified in high amounts can also influence the inhibition of certain skin enzymes (elastases, collagenases and hyaluronidases) which are responsible for the degradation of structural proteins. In addition, they act as mild (2–12 SPF) or moderate (12–30) sunscreens [23]. The main anthocyanins include cyanidin 3-galactoside, which, according to [38], is the most abundant anthocyanin, delphinidin-3-galactoside and pelargonidin-3-galactoside. Moreover dogwood berries contain also a significant levels of iridoids such as cornuside or loganic acid, hydrolyzable tannins, elagic and gallic acid derivatives which are the main antimicrobial and anti-inflammatory agents identified in dogwood fruit. Fresh dogwood berries are rich in vitamin C which is present in quantities twice as high as in oranges [23].

For the purpose of the present study, a technology for obtaining dogwood extracts via solvent extraction was developed. Water and mixtures of water with glycerine, water with trimethylglycine (betaine), and water with plant-derived glycol (propanediol) were used as natural extraction solvents. The extracts thus obtained were assessed to determine their basic biochemical properties, including the ability to neutralize free radicals and the content of polyphenols, anthocyanins and flavonoids. Furthermore, a prototypical natural body wash gel formulation containing the extracts was developed. The skin irritation potential of each product was assessed by determining the zein number and the increase in the pH level of the bovine serum albumin (BSA) solution. Both tests simulate the interaction taking place between the cosmetics under study and the skin proteins. The effect of the extract on the viscosity and foaming ability of the products was also assessed.

## 2. Results and Discussion

Polyphenolic compounds of natural origin constitute a broad group of plant secondary metabolites. Synthesized in small quantities, they are effective UV filters. They protect plants against pests and have powerful antioxidant properties [39]. A significant part of the group are flavonoids and anthocyanins (a subgroup of flavonoids). Dogwood berries are a raw material which is relatively rich in polyphenolic compounds—despite differences in their content depending, among others, on the plant variety or the year of harvest, their average content is estimated at around 370 mg·g^−1^ of fresh weight [26,27,32,33,34].

The extraction of polyphenolic compounds from the plant material is the first step towards their application in the manufacture of cosmetic products. Polyphenols can be extracted with varying effectiveness both from fresh, frozen and dried dogwood berries, and from dogwood leaves [26,27].

The most commonly used method of extracting phenolic compounds is based on solvents. Solvent extraction is possible mainly due to the fact that the process is relatively uncomplicated, highly efficient and broadly applicable [29]. It is widely recognized that the efficiency of chemical extraction depends to a large extent on the type and polarity of extraction solvents used. Other factors impacting the extractability of phenols from the plant material include extraction temperature and time, method of plant material preparation, and raw material to solvent ratio. Since dogwood berries can contain both simple polyphenols and macromolecular polymers, their solubility depends to a large extent on the arrangement and polarity of solvents used. The technique of extracting phenolic compounds from plant materials using water, though not as effective as methods involving other solvents [28], is employed for obtaining raw materials with potential applications in cosmetic products. As shown below, an extraction solvent containing hydrophilic substances which are desirable from the viewpoint of future applications can have a varying capacity to extract polyphenolic compounds from the plant material.

Determination of the content of polyphenolic compounds with the Folin-Ciocalteu reagent demonstrated that an addition of hydrophilic substances in each case increased the ability of the solvent to extract polyphenolic compounds from dogwood berries. Whereas the aqueous extract contained an average of 540.9 milligram equivalents of gallic acid (mgGAe) per litre, the extracts enriched with betaine (BE+D), glycerine (GE+D) and plant glycol (PDE+D) contained 1214.9, 647.0 and 864.1 mgGAe/L, respectively.

**Figure 1:**
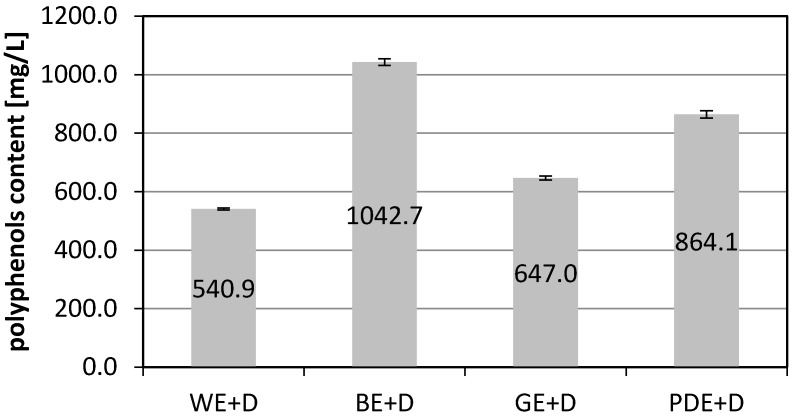
Total content of polyphenols expressed as mg of Gallic acid equivalents (GA) in 1 L of dogwood extracts (WE+D—water extract, BE+D—water/betaine extract, GE+D—water/glycerin extract, PDE+D—water/propanediol extract) averaged from three measurements.

An assay performed for blank extractants samples showed that only the aqueous solution of betaine (BE) yielded a positive sample result. Other extractants did not contained polyphenolic compounds. The content of polyphenolic compounds in BE was determined on the basis of the calibration curve of gallic acid (10–100 mg/L) at the level of 172.2 mgGAe/L (Figure 1).

The presence of polyphenolic compounds in the raw material can be attributed to the content of natural contaminants (polyphenols included) remaining after the process of betaine isolation from plants [30].

Flavonoids are an important group of naturally occurring polyphenolic compounds. Among over 5000 known compounds, six main subclasses—anthocyanidins, flavan-3-ols, flavonols, flavanones, flavones and isoflavones—are significantly involved in human metabolism [40,41]. The ability of the human body to metabolize flavonoid compounds is associated with their bioavailability determined by physical and chemical characteristics. Since the primary metabolism takes place in the digestive system and the liver, the compounds are strongly modified before reaching the skin [42,43,44]. As research shows, topical cutaneous application of flavonoids is an effective method of achieving pharmacological concentrations of flavonoids in the skin [45].

Studies on the optimization of flavonoid extraction from the plant material have demonstrated that microwave and ultrasonic techniques are characterized by considerably higher extraction ability than the traditional method of maceration. At the same time, a change in solvent polarity through an addition of ethyl alcohol has been found to significantly increase the amount of extracted compound [46,47].

Assays performed during the study reported here showed that out of all investigated solvent formulations the highest flavonoid extraction ability was associated with the aqueous solution of glycerine (GE). Flavonoids assayed in the extract made up over 4.1% of all extracted polyphenols. The aqueous extract also contained a significant quantity of flavonoids, amounting to 17.1 mg/L, which constitutes nearly 3.2% of the total quantity of extracted polyphenols.

**Figure 2:**
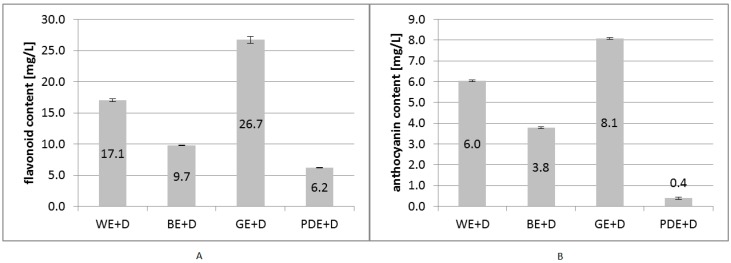
Total content of flavonoids expressed as mg of quercetin equivalents (QU) in 1 L (**A**) and total content of anthocyanins (**B**) in samples of dogwood extracts (WE+D—water extract, BE+D—water/betaine extract, GE+D—water/glycerin extract, PDE+D—water/propanediol extract) averaged from three measurements.

The extracts enriched with betaine and plant glycol were shown to have considerably lower flavonoid extraction ability—both in general and in proportion to extracted polyphenols. The ratio of extracted flavonoids to phenols was found to be 0.93% and 0.72% for the betaine solution and for the plant glycol solution, respectively. It must be noted that none of the solvents under study gave positive results in a reaction determining the content of flavonoids (Figure 2).

Similar tendencies were observed in the analysis of the anthocyanin content in the extracts under study. The highest content of the active compounds was noted for the GE+D extract, and the lowest—for the PDE+D extract (Figure 2).

The results obtained in the study show that the flavonoid extraction ability is proportional to the polarity of the solvent used. These findings are thus consistent with research conducted by other authors [31]. A similar tendency was shown for anthocyanins, which are a subgroup of flavonoids.

The antioxidant properties of the extracts enriched with dogwood berries were investigated by two methods. The antioxidant activity towards the DPPH^•^ radical and the ABTS radical cation was assessed. The extraction of dogwood berries was performed with water (WE+D), and with the addition of a mixture of water and hydrophilic moisturizing substances including betaine, glycerine and propanediol (BE+D, GE+D, PDE+D). The above-mentioned extracts were assessed to determine their antioxidant activity. The test was also performed for blank samples not containing any dogwood berries.

Antioxidant compounds present in dogwood berries included, among others, phenolic compounds, flavonoids, anthocyanins and vitamin C [32,33,47]. Their antioxidant activity is dependent primarily on the number and also the positions of hydroxyl groups in the molecular structure [34,36]. In [37], the level of phenolic compounds has been shown to correlate very well with their antioxidant activity. Similar tendencies were noted in the present study.

The analyses demonstrated the extracts under study to possess varying antioxidant properties. The most potent antioxidant activity—both in the DPPH^•^ and the ABTS methods—was observed in the hydrophilic extracts with dogwood and betaine, and dogwood and propanediol. The analysis of blank samples (water + hydrophilic substance without dogwood) showed that hydrophilic substances alone were characterized by poor antioxidant properties. Extractants showed no ability of free radicals scavenging in concentrations smaller than 1.2 mg·mL^−1^, and in concentrations over 1.2 mg·mL^−1^ the maximal ability of scavenging both (DPPH and ABTS) radicals was assessed on a level at about 8%. At the concentration of 5 and 2.5 mg·mL^−1^, the activity determined for the BE+D and PDE+D extracts amounted to 5%–8% and 2%–4%, respectively. An addition of hydrophilic substances to an extract significantly increases the ability of the solvent to extract antioxidant compounds from dogwood berries. 

**Figure 3:**
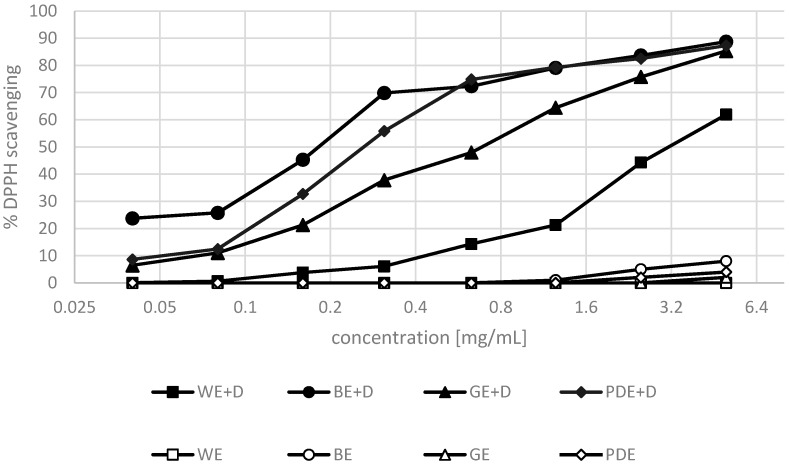
Analysis of antioxidant properties (DPPH methods) in samples of extractants (WE—water, BE—water/betaine, GE—glycerin/betaine, PDE—propanediol/water) and extracts from dogwood fruits (WE+D—water extract, BE+D—water/betaine extract, GE+D—water/glycerin extract, PDE+D—water/propanediol extract) averaged from 3 measurements.

**Figure 4:**
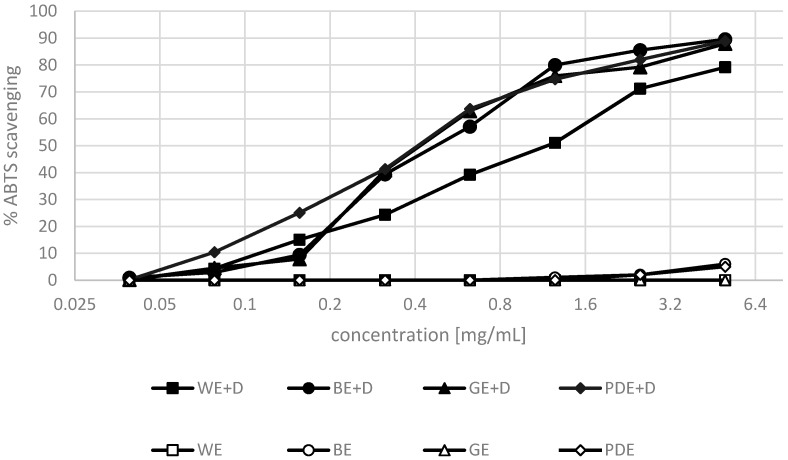
Analysis of antioxidant properties (ABTS methods) in samples of extractants (WE—water, BE—water/betaine, GE—glycerin/betaine, PDE—propanediol/water) and extracts from dogwood fruits (WE+D—water extract, BE+D—water/betaine extract, GE+D—water/glycerin extract, PDE+D—water/propanediol extract) averaged from 3 measurements.

The lowest ability to scavenge the DPPH^•^ radical and the ABTS radical cation was shown for the aqueous extract enriched with dogwood extract (Figure 3 and Figure 4). The above results indicate that dogwood extracts enriched with hydrophilic moisturizing substances have strong antioxidant properties and can be used as a multifunctional raw material in the cosmetic industry.

Safety of use is one of the most important quality attributes applicable to body wash cosmetics. Such products should not produce an adverse impact on the skin by triggering irritations and allergies or causing dry skin. As mentioned in the introductory section, the irritant effect of body wash cosmetics stems mainly from the fact that they contain surfactants which, by interacting with the skin surface proteins, can lead to their denaturation. The prototypical body wash gel formulations developed for the study were subjected to an analysis of their irritant potential based on measuring the zein number and the increase in the pH level of the BSA solution. Both tests imitate the effect of the samples under analysis on proteins. On that basis, it is possible to predict the irritant effect of cosmetic products [1,2,3,4,5].

**Figure 5:**
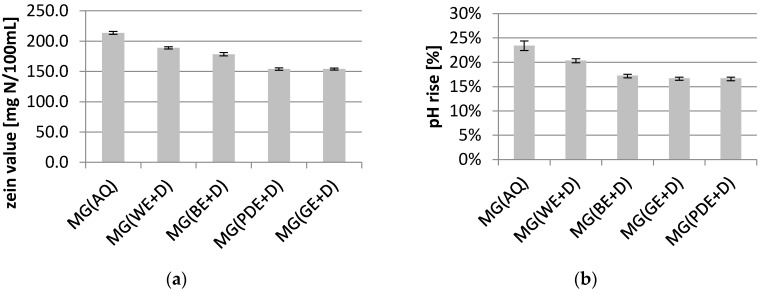
Zein value (**a**) and increase of pH of BSA solution (**b**) of model body wash gels, where: MG(AQ)—model gel containing water in place of extract, MG(WE+D), MG(BE+D), MG(GE+D), MG(PDE+D)—model gels containing 10% (*w*/*w*) of appropriate extracts. The final result was the arithmetic mean of five independent measurements.

The results of the study indicate unequivocally that the addition of hydrophilic dogwood extracts to body wash cosmetic formulations contributes significantly to reducing the skin irritation potential of the products (Figure 5). Measurements of the zein number showed an approx. 15% decrease in the irritant potential, relative to the baseline sample, for the samples containing the aqueous extract and water/betaine extract. A decidedly higher capacity to reduce the skin irritation effect was observed for the samples with the water/glycerine and water/glycol extracts. In their case, the drop in the parameter under study did not differ significantly, and amounted to approx. 28% in relation to the baseline sample. The results of zein number measurements were verified by determining the increase in the pH level of the BSA solution. The results of the determination were consistent with the zein number analysis. The highest pH increase was achieved for the baseline sample (approx. 23%). The addition of extracts contributed to reducing the pH increase, with the lowest values recorded for the MG(BE+D), MG(PG+D) and MG(GE+D) samples (pH increase by approx. 16% relative to the baseline sample). The pH increase determined for the MG(WE+D) sample was approx. 20%.

The literature data show that for 1% of solutions of Sodium Lauryl Sulfate (SLS) the value of the zein number and the increase in the pH level of the BSA solution are approx. 600 mgN/100 mL and 30%, respectively [1,2,3,4,5,48,49]. The prototypical body wash gel formulations were developed with a mixture of an anionic (SLS), amphoteric (Cocamidopropyl Betaine) and a nonionic washing agent (Lauryl Glucoside). The results obtained for the baseline sample confirm the claims made in the literature that using surfactant mixtures influences the stabilization and increase in micelle size, resulting in a decrease of the irritant activity of anionic surfactants [1,2,3,4,5]. The reduction of the skin irritation potential in the samples containing dogwood extracts with solvents and hydrophilic substances can be attributed to stronger micelle stabilization and increase in micelle size in these systems. Hydrophilic molecules can be embedded into the micelle structure, accumulating in the hydrophilic region of the aggregates [50,51,52,53,54,55]. This phenomenon contributes to an increase in the repulsive forces between the hydrophilic parts of surfactant molecules in micelles, leading to a rise in their size. The process can result in a higher capacity to reduce the irritant effect of samples containing hydrophilic extracts. Polar substances and solvents furthermore reduce the CMC of surfactants, which also affects the stabilization of micellar aggregates [50,51,52,53,54,55]. Aside from hydrophilic substances, attenuation of the skin irritation potential can also be achieved with active substances extracted from dogwood berries. The claim is justified by the fact that the irritant potential of the sample containing exclusively the aqueous extract is considerably lower in comparison to the baseline.

**Figure 6:**
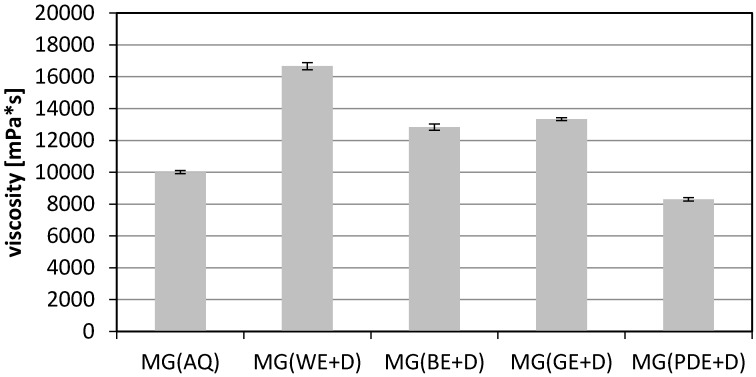
Viscosity of model wash gels, where: MG(AQ)—model gel containing water in place of extract, MG(WE+D), MG(BE+D), MG(GE+D), MG(PDE+D)—model gels containing 10% (*w*/*w*) of appropriate extracts. The final result was the arithmetic mean of five independent measurements.

A very important quality attribute of cosmetic products is viscosity. An appropriately selected viscosity level provides the product with application properties which are considered desirable by consumers and, in their view, make the product more efficient. It was observed during the study that an addition of the studied extracts to the baseline sample had a significant effect on product viscosity. The viscosity level of gels containing the extracts increases in comparison with the extract-free baseline sample. The highest value (ca. 17,200 mPa∙s) was noted for the sample containing the aqueous dogwood extract (MG(WE+D)). At the rotational speed of the measurement spindle amounting to 10 rpm, the sample enriched with the aqueous dogwood extract MG(WE+D) was found to have increased in viscosity by 40% relative to the baseline sample. Lower values were noted for the samples containing the extract with glycerine MG(GE+D) and betaine MG(BE+D) (Figure 6).

**Figure 7:**
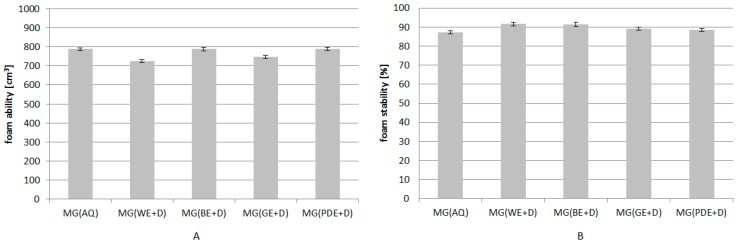
Foam ability (**A**) and foam stability (**B**) of model wash gels, where: MG(AQ)—model gel containing water in place of extract, MG(WE+D), MG(BE+D), MG(GE+D), MG(PDE+D)—model gels containing 10% (*w*/*w*) of appropriate extracts. The final result was the arithmetic mean of five independent measurements.

Based on relevant tests, the effect of the dogwood extract on the foaming properties of body wash gels was evaluated. The data thus obtained give grounds to conclude that the addition of hydrophilic extracts with a higher polarity (WE+D and GE+D) slightly reduces the foaming properties of the products studied. For the extracts with a lower polarity (PDE+D, BE+D), the foaming properties of the samples remained at a level close to the foaming ability determined for the blank sample (Figure 7).

## 3. Material and Methods

### 3.1. Extract Derivation Method

The study was carried out with the *Cornus mas* cultivar Bolestraszycki. Following the removal of seeds, the plant material was dried at the temperature of 40 °C until constant weight was achieved. A total of 5 g of dogwood was used in the study. The material was reduced in size. Natural extraction solvents used in the study included water (WE+D) and mixtures of water with glycerine (GE+D), water with trimethylglycine (betaine) (BE+D) and water with plant-derived glycol (propanediol) (PDE+D). The extracts were prepared in the following ratios: water—79%, hydrophilic moisturizing substance—20%, preservative—1% and dogwood extract—5 g. The extraction process was conducted for 24 h without access of light. The extracts were then decanted and filtered under reduced pressure through filter paper. The extracts were stored in dark glass bottles at the temperature of 4 °C.

### 3.2. Determination of Total Phenolic Content

The determination of total phenolic content in extracts was evaluated spectrophotometrically by the Folin-Ciocalteu method modified by [28]. Respectively, 300 µL of analysed extracts or solvents were mixed with 1500 µL of 1:10 Folin-Ciocalteu reagent and after 6 min in the dark 1200 µL of sodium carbonate (7.5%) was added and samples were incubated in the dark for 2 h. After incubation absorbance at 740 nm was measured (Helios Aquamate spectrophotometer, Thermo Scientific, Waltham, MA, USA). The total phenolic concentration was calculated from a gallic acid (GA) calibration curve (10–100 mg/L). Data were expressed as mg of gallic acid equivalents (GA) in 1 L of extract averaged from three measurements. 

### 3.3. Determination of Flavonoid Content

The total flavonoid content was determined spectrophotometrically by the procedure reported by [56], modified using aluminum nitrate nanohydrate [57]. To prepare a sample 600 µL of appropriate extract or solvent was mixed with 2400 µL of reaction mixture (80% C_2_H_5_OH, 10% Al(NO_3_)_3_ × 9 H_2_O, 1 M C_2_H_3_KO_2_). After 40 min of incubation at room temperature the absorbance was measured at 740 nm using the Helios Aquamate spectrophotometer. The flavonoid content was calculated from a quercetin hydrate calibration curve (10–100 mg/L) and expressed as mg of quercetin equivalents (QU) in 1 L of extract averaged from three measurements.

### 3.4. Determination of Total Monomeric Anthocyanin Pigment Content

The content of total monomeric anthocyanin pigment were assessed by the pH differential method according to [58]. The absorbance of the extract was measured at 520 and 700 nm in buffers at pH 1.0 (hydrochloric acid–potassium chloride, 0.025 M) and 4.5 (acetate acid–sodium acetate, 0.4 M), respectively. Anthocyanin content was calculated as follows:

$$\frac{A\;\times MW\;\times DF\;\times \;10^{3}}{\varepsilon \;\times l}$$

where *A* = (*A*_520nm_ − *A*_700nm_) pH 1.0 − (*A*_520nm_ − *A*_700nm_) pH 4.5; *MW* (molecular weight) = 449.2 g/mol for cyaniding-3-glucoside (cyd-3-glu); *DF* = 1 (dilution factor established during procedure); *l* = 1 (pathlength in cm); ε = 26,900 molar extinction coefficient, in L × mol^−1^ × cm^−1^, for cyd-3-glu; and 10^3^ = factor for conversion from g to mg. An assay was conducted in triplicates. Results were expressed as mg cyanidin-3-glucoside equivalents.

### 3.5. DPPH^•^ Radical Scavenging Activity

DPPH^•^ radical scavenging by dogwood extracts was performed according to [59]. 1 mL of dogwood extract or appropriate solvent was mixed with 1 mL 25 mM DPPH^•^ solution in 96% ethanol. Following 40 min incubation at room temperature the absorbance of the sample was measured at λ = 515 nm using the Helios AquaMate spectrophotometer. 96% ethanol was used as a blank sample. All samples were analyzed in triplicates. The percentage of DPPH^•^ scavenging was calculated for each sample based on the equation:

% of DPPH^•^ scavenging = [1 − (As/Ac)] × 100%

where As—absorbance of the sample; Ac—absorbance of the control sample (DPPH^•^ solution).

### 3.6. ABTS^•+^ Radical Scavenging Activity

Scavenging of ABTS^•+^ free radical was evaluated according to [60] in modification [61]. The scavenging reaction is based on decolorisation of the green ABTS radical cation (ABTS^•+^). To prepare the ABTS^•+^ solution 19.5 mg ABTS and 3.3 mg potassium persulfate was mixed with 7 mL of phosphate buffer pH = 7.4 and dissolved for 16 h in darkness. The solution was diluted to reach the absorbance at λ = 414 nm around 1.0. 20 µL of dogwood extract or appropriate solvent was mixed with 980 µL diluted ABTS^•+^ solution and incubated for 10 min. The decrease in ABTS^•+^ absorbance was measured at λ = 414 nm using the Helios AquaMate spectrophotometer, with distilled water as a blank. All samples were analysed in triplicate. The percentage of ABTS^•+^ scavenging was calculated based on the equation:

% of ABTS^•+^ scavenging = [(1 − (As/Ac)] × 100

where As—absorbance of the sample; Ac—absorbance of the control sample (ABTS^•+^ solution).

### 3.7. Technology for Obtaining Prototypical Body Wash Gels

Based on prior experiences, prototypical body wash gel formulations were developed. The formulations meet the requirements applicable to natural cosmetics. The compositions are listed in Table 1 below.

Overall, five samples of prototypical body wash gels were obtained. The preparation method was the same for all the samples. First, demineralized water and a preservative (Sodium Benzoate and Potassium Sorbate) were measured out into a beaker. The contents of the beaker were heated to the temperature of 60 °C, following which the washing agents (Sodium Lauryl Sulfate, Cocamidopropyl Betaine and Lauryl Glucoside) were added successively. After each substance was added, the contents of the beaker were mixed until the substances dissolved completely, using a magnetic stirrer (propeller mixer, rotational speed 250 rpm). Next, Citric Acid and Sodium Chloride were added as viscosity modifiers. The samples were then cooled, and the previously prepared dogwood extracts were added: aqueous (sample MG(WE+D)), water-glycerine (MG(GE+D)), water-propanediol (MG(PDE+D)) and water-betaine (MG(BE+D)). An extract-free sample (MG(AQ)) was used as a reference sample. Each of the samples was homogenized (IKA homogenizer; 3000 rpm) and left at room temperature until complete air elimination. 

### 3.8. Zein Test

Irritant potential of the products was measured using a zein test. In the surfactants solution zein protein is denatured and then is solubilized in the solution. This process simulates the behaviour of surfactants in relation to the skin proteins. To 40 mL of the sample solution (10 wt %) was added 2 ± 0.05 g of zein from corn. The solutions with zein was shaken on a shaker with water bath (60 min at 35 °C). The solutions were filtered on Whatman No. 1 filters and then centrifuged at 6720 g for 10 min. The nitrogen content in the solutions was determined by Kjeldahl method. 1 mL of the filtrate was mineralized in sulfuric acid (98%) containing copper sulphate penthahydrate and potassium sulphate. After mineralization the solution was transferred (with 50 mL of MiliQ water) into the flask of the Wagner–Parnas apparatus. 20 mL of sodium hydroxide (25 wt %) was added. The released ammonia was distilled with steam. Ammonia was bound by sulfuric acid (5 mL of 0.1 N H_2_SO_4_) in the receiver of the Wagner–Parnas apparatus. The unbound sulfuric acid was titrated with 0.1 N sodium hydroxide. Tashiro solution was used as an indicator. The zein number (ZN) was calculated from the equation:

ZN = (10 − V1) × 100 × 0.7 (mg·N/100 mL)

where V1 is the volume (cm^3^) of sodium hydroxide used for titration of the sample. The final result was the arithmetic mean of five independent measurements.

### 3.9. Determination of Irritant Potential—pH Rise Test with Bovine Albumin Serum (BSA)

50 g of aqueous solution of BSA (2 wt %, pH = 5.5) was mixed with 50 g of body wash gels solution (10 wt %, pH = 5.5). pH of the BSA and analyzed samples solution was regulated with the sodium hydroxide or citric acid. Samples were stirred (200 rpm, 3 h). After 72 h incubation at room temperature pH was measured (pH-meter; Elmetron, Zabrze, Poland) combination pH electrode, 22 °C). The final result was the arithmetic mean of five independent measurements. The results were presented as the difference in pH of the solution after incubation and pH of the solution before incubation (value 5.5).

### 3.10. Viscosity Measurements

A Fungilab Expert (Fungilab, Barcelona, Spain) rheometer was used. Measurements were carried out at 22 °C with a rotary speed of the spindle of 10 rpm. Viscosity values presented in the figures represent average values obtained from five independent measurements.

### 3.11. Determination of the Foaming Properties

The method of measurement was in line with Polish Standard PN-ISO 696:1994P (Surface active agents—Measurement of foaming power—Modified Ross-Miles method). The foam volume produced by 500 mL of samples solutions (1 wt %) falling from a height of 450 mm into a cylinder (1000 mL) containing 50 mL of the same solution was measured. Measurements were carried out at 22 °C. The final result was the arithmetic mean of five independent measurements. Foam stability was calculated from the equation:

Foam stability = V_10_/V_1_ × 100% (1)

where V_10_—foam volume after 10 min, V_1_—foam volume after 1 min.

### 3.12. Error Analysis

The points in the charts represent mean values from a series of three or five independent measurements. The *t*-distribution was used to calculate confidence limits for the mean values. Confidence intervals, which constitute a measuring error were determined for the confidence level of 0.90. Error values are presented in the Figures.

## 4. Conclusions

The study demonstrated that the addition of a dogwood extract to body wash gel formulations was an important factor contributing to their quality. The addition of extracts to formulations contributed significantly to a decrease in body wash gel skin irritation potential and improved the safety of product use. However, no significant effect of the extracts on the foaming properties of the gels was noted. The viscosity increase was observed in cleansing gels containing WE+D, GE+D and BE+D extracts. The introduction of a hydrophilic substance into the extractant was found to enhance the ability of the solvent to extract compounds with antioxidant properties. The extracts under study were characterized by a high content of phenolic compounds, flavonoids and anthocyanins.

The results thus demonstrate that the extracts under study could be used as an innovative and multifunctional raw material in the cosmetic industry. Not only do dogwood extracts significantly decrease the irritation potential of anionic surfactant—based wash gels, but they also contains high contents of bioactive compounds. These properties might be beneficial, not only for cleansing cosmetics, but also in personal care formulations or household chemistry products, e.g. hand dishwashing liquids. The positive interactions between bioactive compounds of dogwood extracts, surfactants and the skin might be very complex. To explain those mechanisms additional assays should be conducted. 

## Figures and Tables

**Table 1:** Formulation of model body wash gels.

Ingredient	Ingredient Content (wt %)				
	MG(AQ)	MG(WE+D)	MG(BE+D)	MG(GE+D)	MG(PDE+D)
Aqua	To 100				
Sodium Lauryl Sulfate	7.0				
Cocamidopropyl Betaine	2.4				
Lauryl Glucoside	2.0				
Citric Acid	0.2				
Sodium Benzoate and Potassium Sorbate	0.9				
Sodium Chloride	1.0				
Cornus Extract (water) WE+D	-	7.0	-	-	-
Cornus Extract (water-betaine) BE+D	-	-	7.0	-	-
Cornus Extract (water-glycerine) GE+D	-	-	-	7.0	-
Cornus Extract (water-propanediol) PPD+D	-	-	-	-	7.0
